# Effect of continuous light on diurnal rhythms in *Cyanothece *sp. ATCC 51142

**DOI:** 10.1186/1471-2164-10-226

**Published:** 2009-05-15

**Authors:** Thanura Elvitigala, Jana Stöckel, Bijoy K Ghosh, Himadri B Pakrasi

**Affiliations:** 1 Department of Electrical and Systems Engineering, Washington University, St. Louis, MO 63130, USA; 2 Department of Biology, Washington University, St. Louis, MO 63130, USA; 3 Department of Mathematics and Statistics, Texas Tech University, Lubbock, TX 79409, USA

## Abstract

**Background:**

Life on earth is strongly affected by alternating day and night cycles. Accordingly, many organisms have evolved an internal timekeeping system with a period of approximately 24 hours. Cyanobacteria are the only known prokaryotes with robust rhythms under control of a central clock. Numerous studies have been conducted to elucidate components of the circadian clock and to identify circadian-controlled genes. However, the complex interactions between endogenous circadian rhythms and external cues are currently not well understood, and a direct and mathematical based comparison between light-mediated and circadian-controlled gene expression is still outstanding. Therefore, we combined and analyzed data from two independent microarray experiments, previously performed under alternating light-dark and continuous light conditions in *Cyanothece *sp. ATCC 51142, and sought to classify light responsive and circadian controlled genes.

**Results:**

Fourier Score-based methods together with random permutations and False Discovery Rates were used to identify genes with oscillatory expression patterns, and an angular distance based criterion was applied to recognize transient behaviors in gene expression under constant light conditions. Compared to previously reported mathematical approaches, the combination of these methods also facilitated the detection of modified amplitudes and phase-shifts of gene expression. Our analysis showed that the majority of diurnally regulated genes, essentially those genes that are maximally expressed during the middle of the light and dark period, are in fact light responsive. In contrast, most of the circadian controlled genes are up-regulated during the beginning of the dark or subjective dark, and are greatly enriched for genes associated with energy metabolism. Many of the circadian controlled and light responsive genes are found in gene clusters within the *Cyanothece *sp. ATCC 51142 genome. Interestingly, in addition to cyclic expression patterns with a period of 24 hours, we also found several genes that oscillate with an ultradian period of 12 hours, a novel finding among cyanobacteria.

**Conclusion:**

We demonstrate that a combination of different analytical methods significantly improved the identification of cyclic and transient gene expression in *Cyanothece *sp. ATCC 51142. Our analyses provide an adaptable and novel analytical tool to study gene expression in a variety of organisms with diurnal, circadian and ultradian behavior.

## Background

Temporal periods of light have provided a significant abiotic selection pressure and greatly influenced evolution on earth. Accordingly, diurnal changes in various biological activities have been observed in different organisms, ranging from bacteria to mammals [1-4]. Central for the capacity to anticipate such environmental changes is an internal clock that controls circadian gene expression and orchestrates cell physiology in synchrony with a day and night cycle [5-8].

Cyanobacteria are the only prokaryotes with a circadian clock [9]. These organisms thrive in environments as diverse as fresh and salt waters, glaciers, deserts, and hot springs, where they play a major role in global carbon sequestration and oxygen production. In addition, some cyanobacteria, such as *Cyanothece *sp. ATCC 51142 (*Cyanothece *51142 hereafter) are able to fix atmospheric nitrogen and contribute to a great extent to the marine nitrogen cycle [10]. *Cyanothece *51142 is a unicellular cyanobacterium that separates oxygenic photosynthesis and nitrogen fixation temporally, thereby circumventing the irreversible inactivation of the oxygen sensitive nitrogenase enzyme [11]. In *Cyanothece *51142, photosynthesis occurs during the day and nitrogen fixation during the night of a diurnal cycle [12].

Previous work has focused on the identification of different clock components such as *kaiA*, *kaiB *and *kaiC*, and several detailed mathematical models describing the interactions between oscillator components have been published [13-15]. Most of these studies are based on modeling transcriptional and translational feedback loops as well as post-transcriptional modifications of clock genes. In other work, global DNA microarray analysis of *Synechocystis *sp. PCC 6803, a non-diazotrophic strain, under constant light conditions determined the extent of circadian control at the transcriptional level, leading to the prediction of 2–7% of cyclic genes [5]. In contrast, microarray analyses of *Cyanothece *51142 under alternating light and dark conditions revealed that ~30% of the genes in the genome are significantly diurnally regulated [16], whereas only 10% of these genes are circadian controlled [17]. However, a detailed analytical approach to distinguish between diurnal and circadian regulated genes in *Cyanothece *51142 is still outstanding.

Organisms such as *Cyanothece *51142 perform different cellular processes at distinct phases of the diurnal cycle and thus depend on tightly regulated expression programs. On the other hand, the capability to adjust to periodic changes in their environment, in particular to alterations of light intensity and quantity, provides an important basis for the ecological success of these organisms. Light is an essential abiotic factor, not only because cyanobacteria obtain their entire energy through photosynthesis, but also for the activation and regulation of many central metabolic processes. In order to understand how *Cyanothece *51142 balances gene expression under nitrogen-fixing conditions in consecutive light and dark cycles with altered physiological requirements under constant light conditions, we analyzed data sets from two different microarray experiments, to elucidate details beyond of what has been found in the former studies [16-18].

For this study we developed various advanced mathematical tools to identify rhythmic and transient patterns of gene expression. We demonstrate that a combination of different analytical approaches substantially improves the distinction between transient and cyclic gene expression. Our analysis revealed that a high percentage of the previously observed strongly diurnally regulated genes [16] are significantly affected under constant light conditions. Interestingly, the majority of circadian controlled genes in *Cyanothece *51142 are maximally expressed during the subjective dark and largely comprised of genes related to energy metabolism. Furthermore, we found a significant number of genes that show ultradian rhythms in their expression and oscillate with a period of 12 hours. This study provides a novel and adaptable analytical tool for comparison of data from different microarray experiments to study gene expression in a variety of organisms with diurnal, circadian and ultradian behavior.

## Results and Discussion

### Microarray data sets

We have analyzed two independent *Cyanothece *51142 microarray data sets that were performed over two consecutive diurnal periods with a sampling rate of every four hours and a shift in sampling time of one hour between the experiments. The studies were conducted using Agilent  custom-made two-channel microarrays. The work of Stöckel *et al*. [16] focused on global gene expression in *Cyanothece *ATCC 51142 under alternating 12 hour light and dark cycles, while Toepel *et al*. [17] investigated changes in gene expression under a 12 hour light and 12 hour dark period followed by a constant light period of 24 hours. In both experiments *Cyanothece *51142 cells were grown under nitrogen-fixing conditions.

### Data analyses and identification of cyclic expressed genes

In Stöckel *et al*. [16] diurnal regulated genes were classified using a correlation network and a fold-change cutoff of 1.3, in which genes with a period other than 24 hours were excluded, since they did not correlate with the main oscillatory network. In Toepel *et al*. [17,18] cyclic genes were identified using a 2-fold cutoff for maximal changes in gene expression. Although the main oscillatory behaviors are detectable using these methods, alterations in gene expression such as changes of amplitudes and phase-shifts were not observed, especially because the altered light conditions were applied only for a short period of time. Therefore we proposed a combination of an angular distance based criteria to characterize such alterations in gene expression.

**Figure 1:**
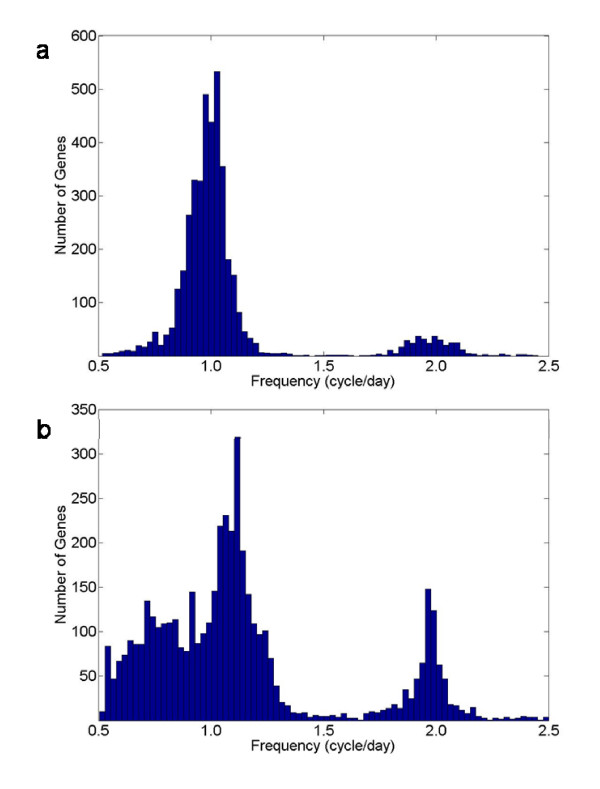
**Distribution of principal frequencies**. Fast Fourier Transform was used to determine the principal frequencies of different gene expressions. (a) Histogram of principal frequencies obtained from the microarray data set under alternating light and dark cycles [16]. (b) Distribution of principal frequencies from the Toepel *et al*. data set [17] under alternating light and dark periods followed by continuous light. The x-axis shows the frequency and the y-axis shows the number of genes.

After normalization of both raw data sets, the main frequency components of gene expression were identified using Fast Fourier Transform (FFT) according to [19]. For the data from Stöckel *et al*. [16], the majority of genes under alternating light and dark cycles oscillate with a period of approximately 24 hours (Figure 1a). Interestingly, a considerable number of genes show a principal frequency close to two cycles per day, which corresponds to genes that oscillate with a period of 12 hours. In comparison, similar analyses of data from Toepel *et al*. [17] show that the frequencies are distributed over a wider range, with a larger number of genes cycling with 12 and 24 hour periods (Figure 1b). The extended periods of 40–48 hours (frequencies between 0.5 and 1 cycles per day) are mainly assigned to genes that do not continue to oscillate under continuous light conditions. Thus, the differences between the two histograms primarily reflect the impact of continuous light on gene expression in *Cyanothece *51142.

The two main frequencies of 12 and 24 hours were used for further Fourier score calculations. For the determination of cyclic behavior, a Fourier score cutoff of 6.5 corresponding to a False Discovery Rate of 2% under alternating light and dark conditions and of 3% under continuous light was used. Based on this analysis, 659 genes that oscillate with a period of 24 hours and 9 genes that cycle with a period of 12 hours were identified as common in both microarray datasets. An additional 1551 genes that oscillate with a period of 24 hours and 83 with a period of 12 hours were detected only in the Stöckel et.al data set. All of these genes were used for further analyses (see below).

### Classification of light responsive genes

**Figure 2:**
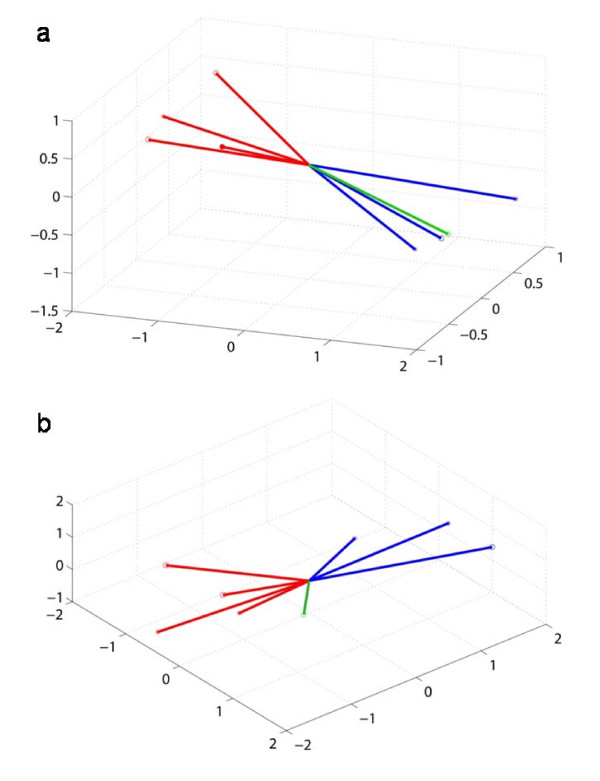
**Distribution of vectors corresponding to different light and dark periods for two distinct hydrogenase genes**. The vectors represent the log_2 _ratios of three time points for each corresponding 12 hour period. The vectors were plotted in a three-dimensional space, with axes corresponding to the 1^st^, 2^nd^, and the 3^rd ^time points within a 12 hour period. (a) The gene *cce_2318 *shows a cyclic expression profile under alternating light and dark conditions and does not reveal significant changes during the subjective dark period. (b) The expression of the gene *cce_1063 *alters considerably under subjective dark. *Red *colored vectors represent the different light periods, *Blue *colored vectors correspond to the dark periods, and *Green *vectors indicate subjective dark.

Since the Fourier score-based method is primarily designed for the identification of cyclic gene expression, and because genes with altered expression patterns are detectible only in time points corresponding to the subjective dark, an angular distance based method was proposed to identify transient behaviors in gene expression. Accordingly, the data were separated into four 12 hour subsets, which correspond to the individual light and dark periods and thus resulted in four 3-dimensional vectors. If a gene shows a cyclic expression, smaller distances between vectors related either to two light or two dark periods are expected. In contrast, larger distances are anticipated for vectors from different periods such as light and dark. For genes showing transient behavior in their expression, the vector equivalent to the subjective dark is likely to be different from the dark (Table 1). Figure 2a shows the distribution of vectors for the gene *cce_2318*, which encodes HoxF, one of the subunits of the bidirectional hydrogenase, as an example of circadian gene expression. All vectors corresponding to individual light and dark periods are well separated and the vector representing the subjective dark is closely associated with the vectors derived from other dark periods. In contrast, the gene *cce_1063*, encoding the small subunit HupS of the uptake hydrogenase, does not continue to oscillate under constant light conditions (Figure 2b). The altered expression of *cce_1063 *is clearly indicated in that the vector related to the subjective dark period shows a different orientation.

**Table 1:** Average distance from different light and dark periods in each of the four classified gene groups.

	**Stöckel *et al*. data set **[16]						**Toepel *et al*. data set **[17]					
**Group**	D_1_L_1_	D_1_D_2_	D_1_L_2_	L_1_D_2_	L_1_L_2_	D_2_L_2_	L_1_D_1_	L_1_L_2_	L_1_SD_2_	D_1_L_2_	D_1_SD_2_	L_2_SD_2_
**1**	1.78	0.11	1.79	1.85	0.08	1.79	1.79	0.12	1.68	1.70	0.33	1.69
**2**	1.78	0.17	1.72	1.88	0.14	1.76	1.73	0.18	1.57	0.68	1.42	0.78
**3**	0.23	0.44	0.29	0.37	0.05	0.44	0.58	0.17	0.27	0.46	0.68	0.27
**4**	0.33	0.17	0.29	0.43	0.20	0.30	1.44	0.40	0.63	1.26	1.04	0.92

D: dark; L: light; SD: subjective dark

**Figure 3:**
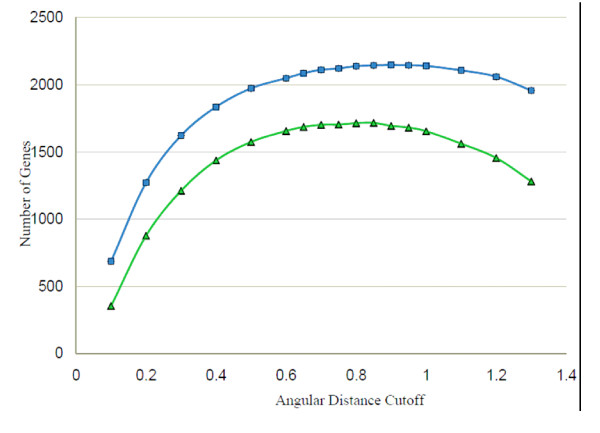
**Threshold evaluation**. The number of cyclic expressed genes using different cutoffs for the angular distance method are shown. The threshold value resulting in the maximum number of oscillating genes for both microarray data sets was chosen for the final classification. The blue graph corresponds to the cyclic genes from the Stöckel *et al*. data set [16] and the green graph shows the oscillating genes from the Toepel *et al*. data set [17].

The similarity threshold chosen for the angular distance method ought to yield a dataset of cycling genes in utmost agreement between the Fourier score based and angular distance methods. Therefore, for the angular distance method the number of cyclic genes among the set of already identified cycling genes was calculated for different threshold values (Figure 3). Based on these calculations, a cutoff of 0.8 for the angular distance was selected, which resulted in 97% and 78% Fourier score based agreement for the Stöckel *et al*. [16] and Toepel *et al*. [17] data sets, respectively. The expression of a gene in two different 12 hour periods was considered to be similar if the corresponding vectors were within a distance of 0.8 to each other, and disparate if the distance was higher. The vectors of genes transcribed with an ultradian period of 12 hours revealed consistently smaller angular distances for any two regions (Table 1).

Based on the Fourier score and angular distance based methods, four major groups of genes could be identified. The genes in group 1 continued to oscillate under continuous light and were classified as circadian controlled genes, whereas genes from group 2 were light responsive and showed oscillating expression only under alternating light and dark cycles. Both groups represent genes that cycle with a 24 hour period. In contrast, genes from group 3 and 4 oscillate with an ultradian period of about 12 hours, with genes from group 3 being circadian controlled and the genes from group 4 light responsive (Table 1, [see Additional files 1, 2]).

**Figure 4:**
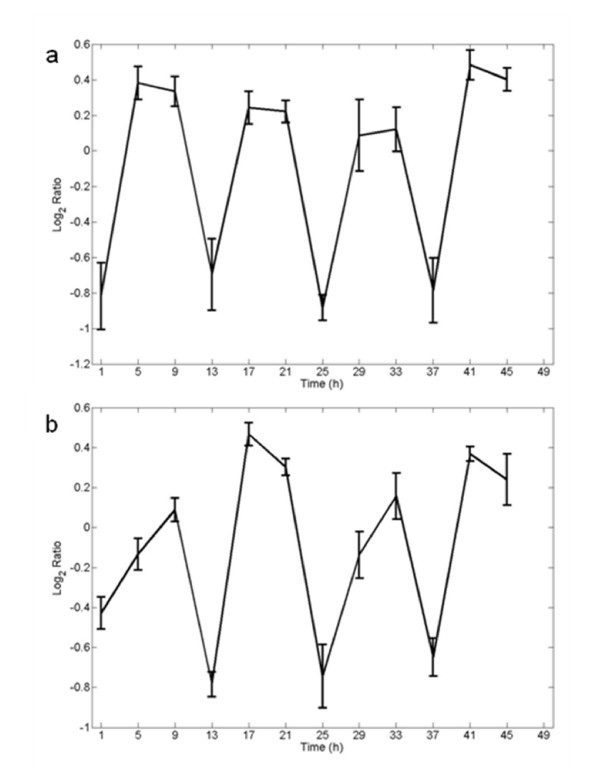
**Ultradian cycles in gene expression**. The genes (a) *cce_1889 *and (b) *cce_3225 *reveal ultradian rhythms in their gene expression and oscillate with a period of 12 hours.

After the application of both criteria, our analysis of the Stöckel *et al*. dataset [16] identified 43% of genes in the *Cyanothece *51142 genome (2138 genes) with oscillating expression patterns under alternating light and dark conditions, in comparison to the previously reported 1445 genes. This suggests that diurnal regulation of gene expression in *Cyanothece *51142 might be greater than previously thought. However, after combining and analyzing both data sets using the two different methods, only 722 (14.8%) of genes in the genome were found to be diurnally regulated or light inducible, and 448 genes (9.2%) could be classified as circadian controlled. This relatively small number of diurnally regulated genes common in both data sets results from the stringent criteria that were used for the gene classification to correct for differences in growth and culture conditions and therefore comprises the more significant cyclic expressed genes. Interestingly, five circadian controlled genes and 45 genes with transient expression patterns oscillate with an ultradian frequency of 12 hours [see Additional file 2]. As an example, figure 4 shows the expression pattern for the genes *cce_1889 *and *cce_3225 *that oscillate with a 12 hour period.

Taken together, the combination of the angular distance and Fourier Score based methods lent an additional level of confidence to the identification of cyclic expressed genes in *Cyanothece *51142. These analyses uncovered that most of the previously identified diurnal genes are indeed light responsive.

### Diurnal regulated genes belonging to different functional categories

Considering the central role of photosynthesis in the cellular metabolism of *Cyanothece *51142 and other cyanobacteria, a great impact of light on gene expression was anticipated. We found that the expression of the majority of regulatory genes is light responsive, presumably the result of comprehensive changes that take place in order to adjust to altered light conditions [see Additional file 1]. In addition, the expression of many genes related to transcription and translation is light regulated and likely accounts for a higher turnover of proteins under continuous light. Our analyses also revealed that several genes associated with photosynthesis, especially with photosystem II (PSII) such as *psbE*, *psbF*, and *psbJ *are circadian controlled [see Additional file 1]. The PsbJ protein in *Synechocystis *sp. PCC 6803 has been shown to control the amount of functionally assembled PSII complexes in the thylakoid membrane [20]. Moreover, *psbE *and *psbF *which encode the α and β subunits of cytochrome b559 are required to stabilize the reaction center of PSII [21]. Interestingly, a number of subunits of the ATP-synthase complex, including AtpF and AtpH, which are encoded by multicopy genes revealed that one gene copy is light responsive while the other gene copy was found to be circadian controlled [see Additional file 1]. The gene *atpH *has been identified as clock controlled in previous studies of *Synechocystis *sp. PCC 6803 [5].

A more detailed analysis of different metabolic processes revealed that nitrate and sulfate assimilation are largely under circadian control [see Additional file 1]. Furthermore, the genes *hoxEFUYH *encoding the bidirectional hydrogenase show a significant cyclic behavior in their expression [see Additional files 3ab. In comparison, a recent microarray analysis performed in *Cyanothece *51142 over 24 hours under alternating 6 hours light/dark conditions [18] led the authors to the conclusion that the expression of the *hox *genes under their conditions are not circadian. However, an alternative interpretation of their data (Figure 3B in [18]) suggests that the *hoxE *gene does in fact show an oscillatory expression with a period of 24 hours, which would support our findings. This is also supported by previous studies in *Synechococcus *sp. PCC 7942 and *Synechocystis *sp. PCC 6803 which uncovered a strong circadian component in the expression of different *hox-genes *[5,22]. In total, our study revealed that 9 out of 17 genes involved in hydrogen metabolism in *Cyanothece *51142 were expressed at the same level during the dark and subjective dark and therefore classified as circadian controlled. In contrast, the expression of *hupS *and *hupL *encoding subunits of the uptake hydrogenase are severely affected during the subjective dark, with *hupS *showing an 8-time lower expression level (see Additional files 3C,D). Earlier studies of different *Nostoc *species uncovered that the expression of *hupL *is substantially stimulated by supplemented hydrogen [23,24]. Accordingly, the reduced transcript abundance of *hupS *and *hupL *is presumably coupled to a decline in the activity of the nitrogenase enzyme, which has been observed during the subjective dark period under continuous light conditions [17]. Furthermore, even though the genes involved in nitrogen fixation are organized in a single cluster consisting of two adjacent regulons on opposite strands in the genome [25], different expression profiles for many of those genes were observed under continuous light. The genes *nifB*, *nifS*, *nifE*, *nifN*, *nifX *and *nifW*, which are involved in biogenesis and assembly of the Mo-Fe cofactor, revealed a more than two fold increase in their expression levels [17] in addition to a shift in phase of about four hours under continuous light conditions [see Additional files 4ab *vs*. 4cd].

In fact, several light responsive and circadian controlled genes are found in clusters of three or more genes throughout the *Cyanothece *51142 genome [see Additional files 1, 5 and 6]. Such clustering of similarly regulated genes into different regions of the genome might provide an opportunity for the cell to control the gene expression more efficiently. On the other hand, additional diurnal and circadian controlled genes can be identified which might not have been detected due to low amplitudes of oscillations. Thus, there is a high probability that an unclassified gene within a group of genes assigned to one of the two categories belongs to the same class.

**Figure 5:**
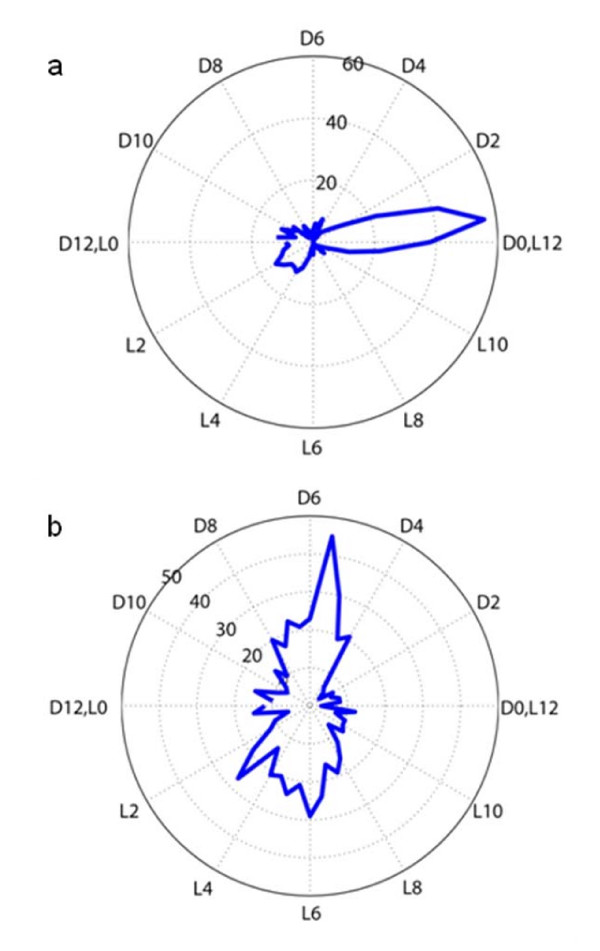
**Peak time distribution for circadian controlled and light responding genes**. The graphs represent the peak time data for genes which were classified as (a) circadian controlled and (b) light responsive. The circle defines a polar coordinate plot with radians transformed to hours. The inner dotted circles represent a scale for the number of genes. The time points are plotted around the circle. Most of the circadian genes peak in the early dark, whereas light responding genes peak in the middle of either light or dark.

Furthermore, we found that the majority of circadian controlled genes are maximally expressed at the beginning of the dark period and are largely comprised of genes related to energy metabolism (Figure 5a). In contrast, most of the light responsive genes peak during the middle of the dark or light periods (Figure 5b), which suggests that their expression is regulated by the presence or absence of light rather than in anticipation of the different light cycles. In addition, FFT, Fourier Score, and angular distance calculations revealed 50 genes that show ultradian rhythms in their expression and oscillate with a period of 12 hours. Ultradian rhythms of cell division have been observed in several organisms including cyanobacteria [26], even though the major timing system for all light-sensitive organisms is a circadian clock that operates with a period of approximately 24 hours. Therefore, the finding of 12 hour cycles in the expression of genes related to other cellular processes was unanticipated and is novel among cyanobacteria. Previous studies in the anoxygenic photosynthetic bacteria *Rhodobacter sphaeroides *and *Rhodospirillum rubrum *uncovered a rhythmicity of gene expression and enzyme activities with a period length of either 12 or 24 hours depending on the growth conditions [27,28]. Studies in eukaryotic unicellular microbes such as yeast have shown that an ultradian oscillator regulates cyclic respiratory activity and global gene expression [29,30]. This is particularly interesting since *cydB*, which encodes a subunit of one of the terminal oxidases in *Cyanothece *51142, shows an ultradian expression in both microarrays datasets, whereas the expression of the majority of respiratory genes is considerably affected under subjective dark conditions [17]. However, further studies are required to elucidate the role and physiological significance of such ultradian rhythms in *Cyanothece *51142.

## Conclusion

In this work we compared two independent microarray experiments to study changes in gene expression under alternating and constant light conditions in *Cyanothece *51142. Two mathematical approaches were applied to differentiate transient behaviors from cyclic gene expression. Fourier score based methods can successfully be used for identification of cyclic behaviors but they fail to detect some transient behaviors such as shifts of the expression peaks or smaller amplitudes under constant light conditions. Therefore, an angular distance based method was applied which improved the detection of such changes. These methods are valid for distinguishing transient from cyclic behaviors even for the limited number of time points in one of the different light treatments. This approach provides an adaptable tool for future studies of diurnal, circadian and ultradian rhythms in various organisms. Furthermore, the combined analysis of two different microarray data sets enabled the extraction of novel details about diurnal and circadian regulation in *Cyanothece *51142 which would not be obtained from the individual experiments alone.

## Methods

### Experimental datasets

For the Stöckel *et al*. microarray data set [16], *Cyanothece *51142 cultures were grown in ASP2 medium without NaNO_3 _[11] at 30°C with air bubbling in alternating 12 hour light and dark cycles with 50 μmol photons/m^2^*s of white light. After 7 days, 150 mL samples were collected every 4 hours for 2 days, starting with 1 hour into the dark period (time point D1). In total, 12 samples were collected.

For the Toepel *et al*. data set [17], *Cyanothece *51142 cultures were grown in an airlift bioreactor (6L BioFlo 3000; New Brunswick Scientific, Edison, NJ) in ASP2 medium without NO_3 _at 30°C in 12 hour light and 12 hour dark cycles. The culture was illuminated by two light-emitting-diode panels using orange (640 nm) and red (680 nm) light, yielding an intensity of 100 μmol photons/m^2^*s inside the bioreactor. The cultures were grown for 5–6 days under alternating light and dark conditions prior to collecting samples for every 4 hours over a time period of 2 days, starting at the transition from dark to light (time point L0) for 24 hours followed by 24 hours of continuous light.

### Algorithms and data processing

#### Preliminary Data Processing

In previous studies [16-18], the data were processed and analyzed differently, which made a direct comparison of the results difficult. Therefore, in this communication both data sets have been combined and analyzed uniformly. The raw data were normalized using the LOWESS normalization algorithm to avoid a systematic intensity based bias which is commonly observed with two-channel microarrays [31] and preliminary data processing of both data sets was performed according to [16].

### Identification of Cyclic Genes using Fourier Score

In previous studies [32], several cycle detection methods were compared and the Fourier score based method with random permutations was shown to be more appropriate for situations where data were available only for a few cycles. The Fourier score for any given time course signal *x*_*i *_at a given frequency *f *was defined as:



where *ω *= 2*πf*.

Initial Fast Fourier Transform calculations indicated existence of oscillations with 24 h and 12 h periods. Therefore Fourier score calculations were performed using those frequencies.

If a given signal consists of a dominant cyclic component of the corresponding frequency, a larger Fourier score is expected. The significance of the Fourier score was quantified by comparing the Fourier score of the original signal with scores of large collection of random signals. These random signals were obtained by using different permutations of the original signal. The original gene expressions were scaled to have a unit standard deviation, which enabled direct comparisons of Fourier scores from different genes.

A False discovery rate (FDR) based approach similar to [33] was used to determine a global threshold for the Fourier scores, in order to separate cyclically expressed genes from transiently expressed genes. An empirical FDR for the Fourier score at a chosen threshold *t *was defined as:



Hereby corresponds *M *to the number of permutations which were used for the null hypothesis. M = 10,000 were chosen for all calculations. *N *represents the total number of genes, *F *the Fourier score for a random signal *j *which was obtained for a gene *k*, and *Fj *the Fourier score of the original expression signal for gene *k*. *I(x) *represents an indicator function as follows:



#### Identification of transient behaviors using angular distance

We propose an angular distance based method to classify the transient gene expression under constant light conditions. The data were separated into four 12 hour data sets, which correspond to the different light and dark periods and resulted in four 3-dimensional vectors for each gene and for each experiment. The pair wise angular distances between different vectors for a given gene were calculated as:



*where x*_1 _and *x*_2 _are the vectors from two different 12 hour periods. *D*_1,2 _can have any value between 0 and 2, with 0 representing vectors with the same direction and 2 representing vectors with the opposite direction.

## Authors' contributions

T.E., J.S., B.K.G. and H.B.P. designed research; T.E. and J.S. performed data analysis; T.E. and J.S. wrote the paper. All authors read and approved the final manuscript.

## Supplementary Material

Additional file 1 **Dataset of cyclic and light responsive genes with a principal frequency of 24 hours**. Number of circadian and light regulated genes. *ORFs are labeled according to the deposited genome sequence in GenBank (Welsh et al., 2008). ^†^Annotations were manually curated, based on sequence homology to other known proteins/domains. Click here for file

Additional file 2 **Dataset of cyclic and light responsive genes with a principal frequency of 12 hours**. Number of circadian and light regulated genes that oscillate with a period of 12 hours. *ORFs are labeled according to the deposited genome sequence in GenBank (Welsh et al., 2008). ^†^Annotations were manually curated, based on sequence homology to other known proteins/domains. Click here for file

Additional file 3 **Expression profiles of genes encoding bidirectional and uptake hydrogenase enzymes**. Expression profiles of genes related to hydrogen metabolism over a period of 48 hours in alternating 12 hour light-dark (a, c) and 24 hours in light-dark followed by 24 hours of continuous light (b, d). The expression of the genes *cce_2315*, *cce_2316*, *cce_2317*, *cce_2318 *and *cce_2319 *that encode subunits of the bidirectional hydrogenase as well as the expression of *cce_2879*, *cce_2902*, *cce_2903*, *cce_2907 *is circadian regulated (a, b). The genes encoding the uptake hydrogenase *cce_1063 *and *cce_1064 *as well as the gene *cce_0951 *were classified as light responsive genes (c, d). Click here for file

Additional file 4 **The expression of nitrogen fixation related genes**. Expression profiles of genes associated with nitrogen fixation over 48 hours of alternating 12 hours light-dark conditions (a, c) and 24 hours of light-dark followed by 24 hours of continuous light (b, d). The expression of *cce_0547*, *cce_0548*, *cce_0549 *and *cce_0560 *is under circadian control (a, b), while the genes *cce_0554*, *cce_0557*, *cce_0563*, *cce_0564*, *cce_0565 *and *cce_0568 *were classified as light responsive (c, d). Click here for file

Additional file 5 **Cluster of nitrogenase related genes in the genome of *Cyanothece *ATCC 51142 and their classification according to the expression profiles under different light conditions**. *White *colored arrows represent genes that are not classified as circadian or light responsive. *Grey *collored arrows indicate genes, that are regulated in response to light and *black *colored genes correspond to circadian controlled genes. Click here for file

Additional file 6 **Location and distribution of light and circadian regulated genes on the circular chromosome of *Cyanothece *51142**. *Green *colored bars represent light responsive genes and circadian controlled genes are shown in *red*. Click here for file
