# Supplementary figures and images for: Effect of continuous light on diurnal rhythms in Cyanothece sp. ATCC 51142
Source: BMC Genomics. 2009 May 15;10:226. doi: 10.1186/1471-2164-10-226 (PMC2695482; doi:10.1186/1471-2164-10-226)

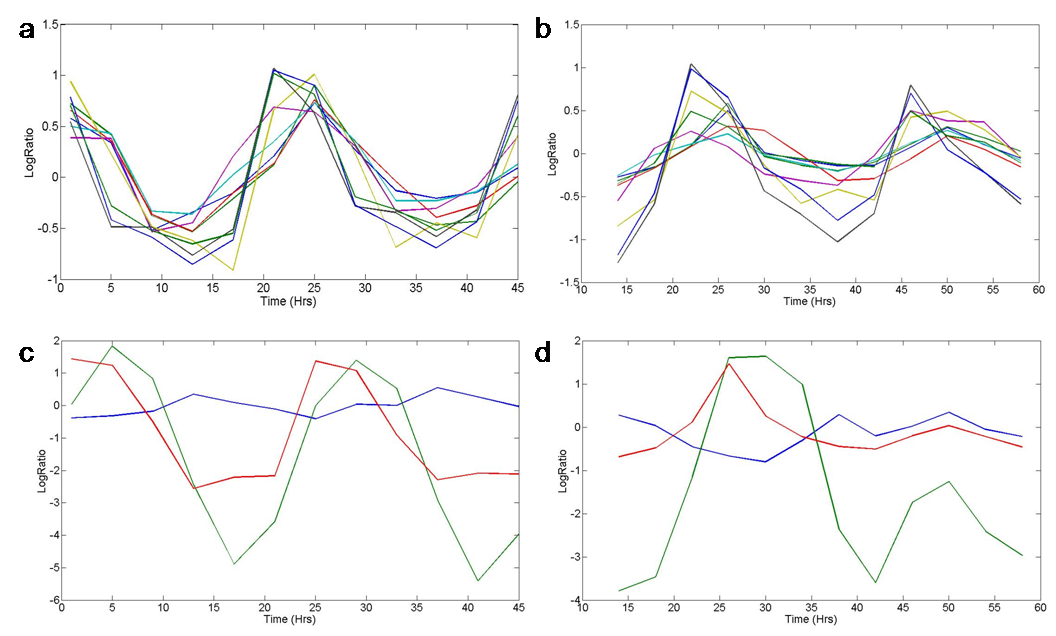

Supplement: Additional file 3 — Expression profiles of genes encoding bidirectional and uptake hydrogenase enzymes. Expression profiles of genes related to hydrogen metabolism over a period of 48 hours in alternating 12 hour light-dark (a, c) and 24 hours in light-dark followed by 24 hours of continuous light (b, d). The expression of the genes cce_2315, cce_2316, cce_2317, cce_2318 and cce_2319 that encode subunits of the bidirectional hydrogenase as well as the expression of cce_2879, cce_2902, cce_2903, cce_2907 is circadian regulated (a, b). The genes encoding the uptake hydrogenase cce_1063 and cce_1064 as well as the gene cce_0951 were classified as light responsive genes (c, d). [file 1471-2164-10-226-S3.doc]

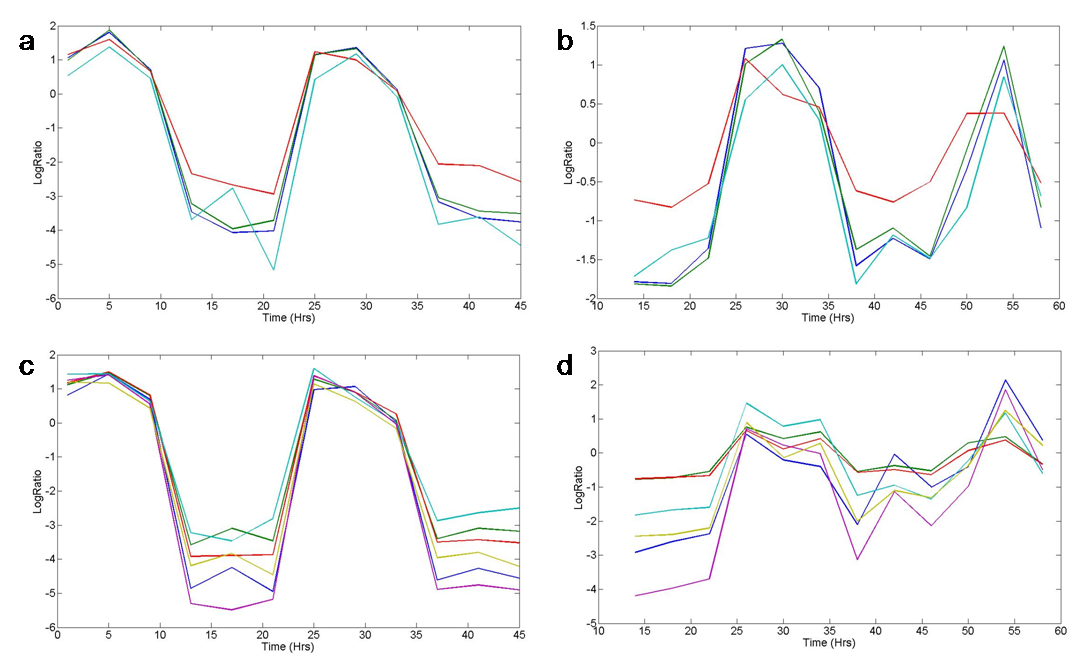

Supplement: Additional file 4 — The expression of nitrogen fixation related genes. Expression profiles of genes associated with nitrogen fixation over 48 hours of alternating 12 hours light-dark conditions (a, c) and 24 hours of light-dark followed by 24 hours of continuous light (b, d). The expression of cce_0547, cce_0548, cce_0549 and cce_0560 is under circadian control (a, b), while the genes cce_0554, cce_0557, cce_0563, cce_0564, cce_0565 and cce_0568 were classified as light responsive (c, d). [file 1471-2164-10-226-S4.doc]

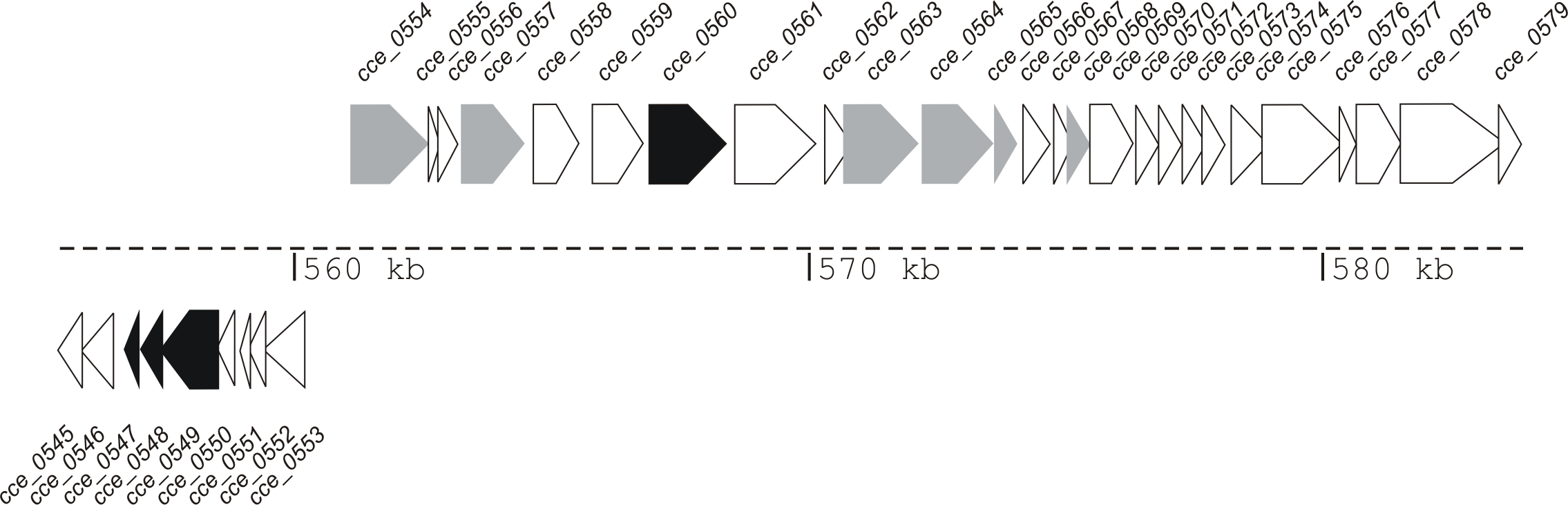

Supplement: Additional file 5 — Cluster of nitrogenase related genes in the genome of Cyanothece ATCC 51142 and their classification according to the expression profiles under different light conditions. White colored arrows represent genes that are not classified as circadian or light responsive. Grey collored arrows indicate genes, that are regulated in response to light and black colored genes correspond to circadian controlled genes. [file 1471-2164-10-226-S5.doc]

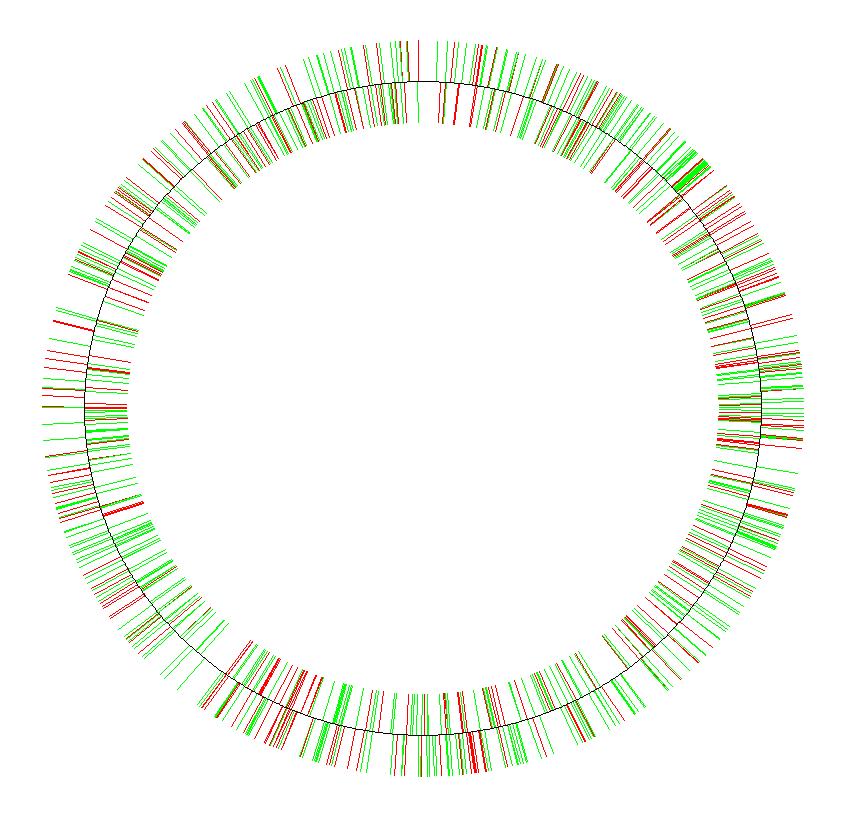

Supplement: Additional file 6 — Location and distribution of light and circadian regulated genes on the circular chromosome of Cyanothece 51142. Green colored bars represent light responsive genes and circadian controlled genes are shown in red. [file 1471-2164-10-226-S6.doc]
